# Supplementary material for: Immunostimulatory Effect of Flagellin on MDR-Klebsiella-Infected Human Airway Epithelial Cells
Source: Int J Mol Sci. 2023 Dec 25;25(1):309. doi: 10.3390/ijms25010309 (PMC10778885; doi:10.3390/ijms25010309)
Supplement: Supplementary file 1 [file ijms-25-00309-s001.zip › ijms-2768742-supplementary.pdf]

Immunostimulatory Effect of Flagellin on MDR-Klebsiella-Infected Human Airway Epithelial Cells

Christine C. A. van Linge [1,2,*], Katina D. Hulme [3], Hessel Peters-Sengers [1,2], Jean-Claude Sirard [4], Wil H. F. Goessens [5], Menno D. de Jong [3], Colin A. Russell [3,6], Alex F. de Vos [1,2] and Tom van der Poll [1,2,7]

Supplemental figures

[1] Center for Experimental and Molecular Medicine, Amsterdam University Medical Centers, University of Amsterdam, 1012 WP Amsterdam, The Netherlands
[2] Amsterdam Infection & Immunity Institute, 1105 AZ Amsterdam, The Netherlands
[3] Department of Medical Microbiology & Infection Prevention, Amsterdam University Medical Centers, University of Amsterdam, 1012 WP Amsterdam, The Netherlands
[4] Center for Infection and Immunity of Lille, Institut Pasteur de Lille, INSERM U1019, CNRS UMR9017, CHU Lille, University Lille, 59000 Lille, France
[5] Department of Medical Microbiology and Infectious Diseases, Erasmus University Medical Center, 3015 GD Rotterdam, The Netherlands
[6] Department of Global Health, School of Public Health, Boston University, Boston, MA 02215, USA
[7] Division of Infectious Diseases, Amsterdam University Medical Centers, University of Amsterdam, 1012 WP Amsterdam, The Netherlands
* Correspondence: c.c.vanlinge@amsterdamumc.nl

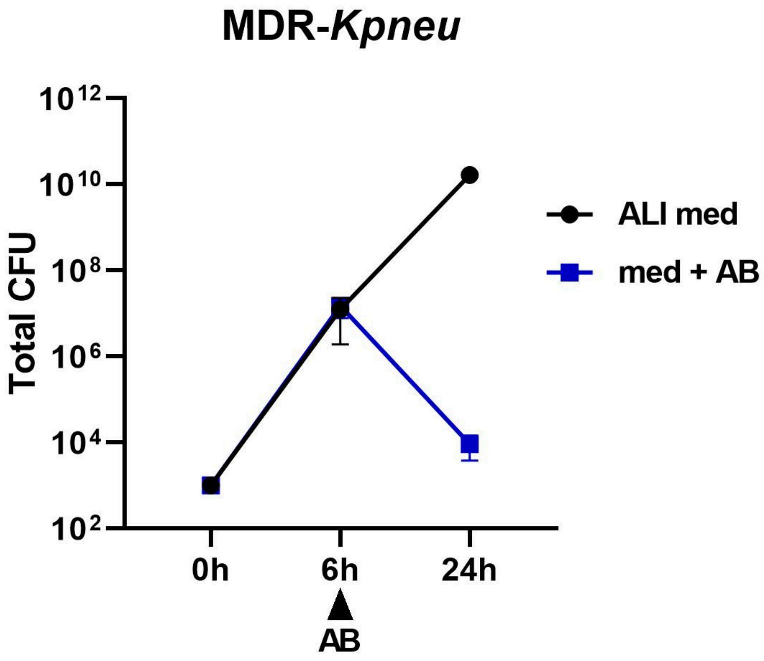

Figure S1. Effect of meropenem on outgrowth of MDR-*Kpneu* in ALI medium. ALI medium was inoculated with 1,000 CFU of log-phase MDR-*Kpneu* and incubated at 37°C and 5% CO_2. After 6 hours, meropenem (AB) was added at a concentration of 50 µg/ml (> 6-fold of the MIC). Bacterial counts were determined at 6 and 24 hours after inoculation.

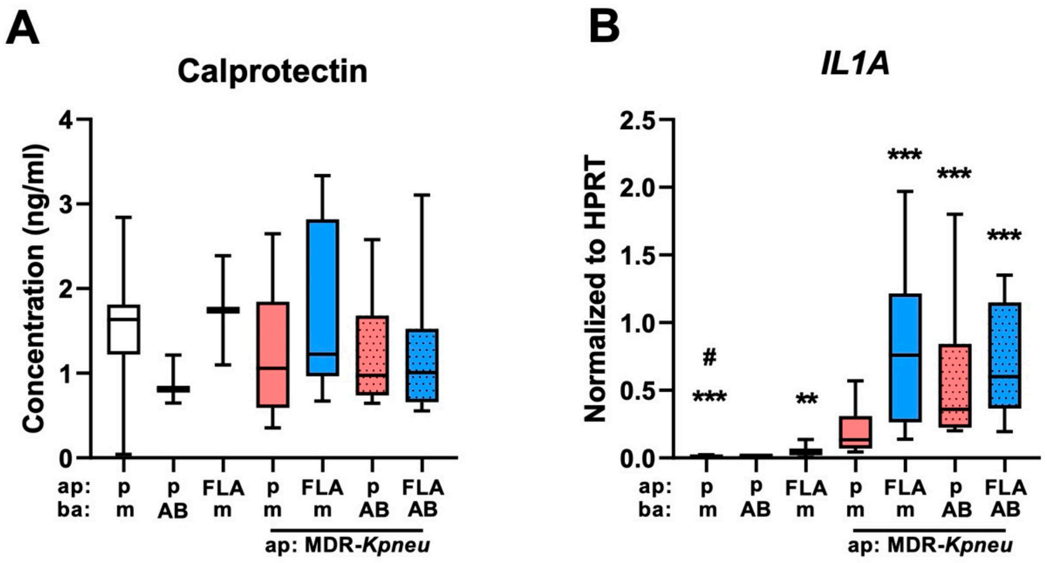

Figure S2. Effect of flagellin on Calprotectin secretion and IL1α expression by MDR-*Kpneu* infected HBE cells. HBE cells were infected on the apical (ap) side with 1,000 CFU MDR-*Kpneu*, treated 6 hours later with 50 μg/ml meropenem (AB) or medium (m) in the basolateral (ba) medium and 0.1 μg flagellin (FLA) or PBS (P) on the apical side. Samples were collected 24 hours after infection. Protein levels of calprotectin (S100A8/A9 heterodimer) in basolateral medium (A). mRNA levels of IL-1α (*IL1A)* relative to the housekeeping gene *HPRT* in HBE cells (B). Data are shown as box and whiskers representing 3 repeated experiments with n=4 per group. # indicate significant differences compared to FLA alone; * indicate significant differences compared to MDR-*Kpneu* and medium treated cells. */#P<0.05, **/##P<0.01, ***/###P<0.001.

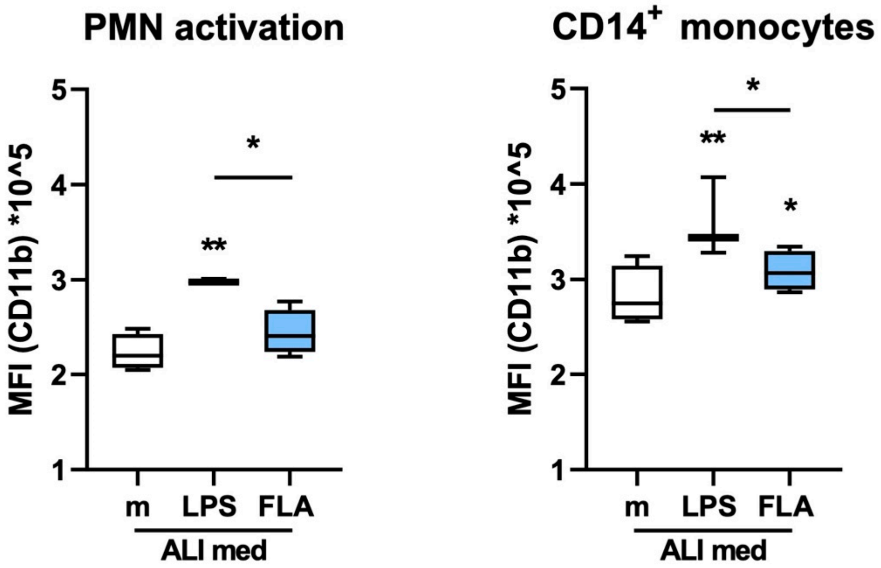

Figure S3. Activation of PMNs and CD14⁺ monocytes in whole blood. Plain ALI medium (m), supplemented with LPS (10 ng/ml) or flagellin (FLA; 1 µg/ml), was incubated with an equal volume of heparinized whole blood for 6 hours after which activation (CD11b mean fluorescence intensity (MFI)) was determined on PMN and $CD14^+$ monocytes by flow cytometry. Data are shown as box and whiskers representing data from 4 healthy blood donors with 4 technical replicates per group *P<0.05, **P<0.01.

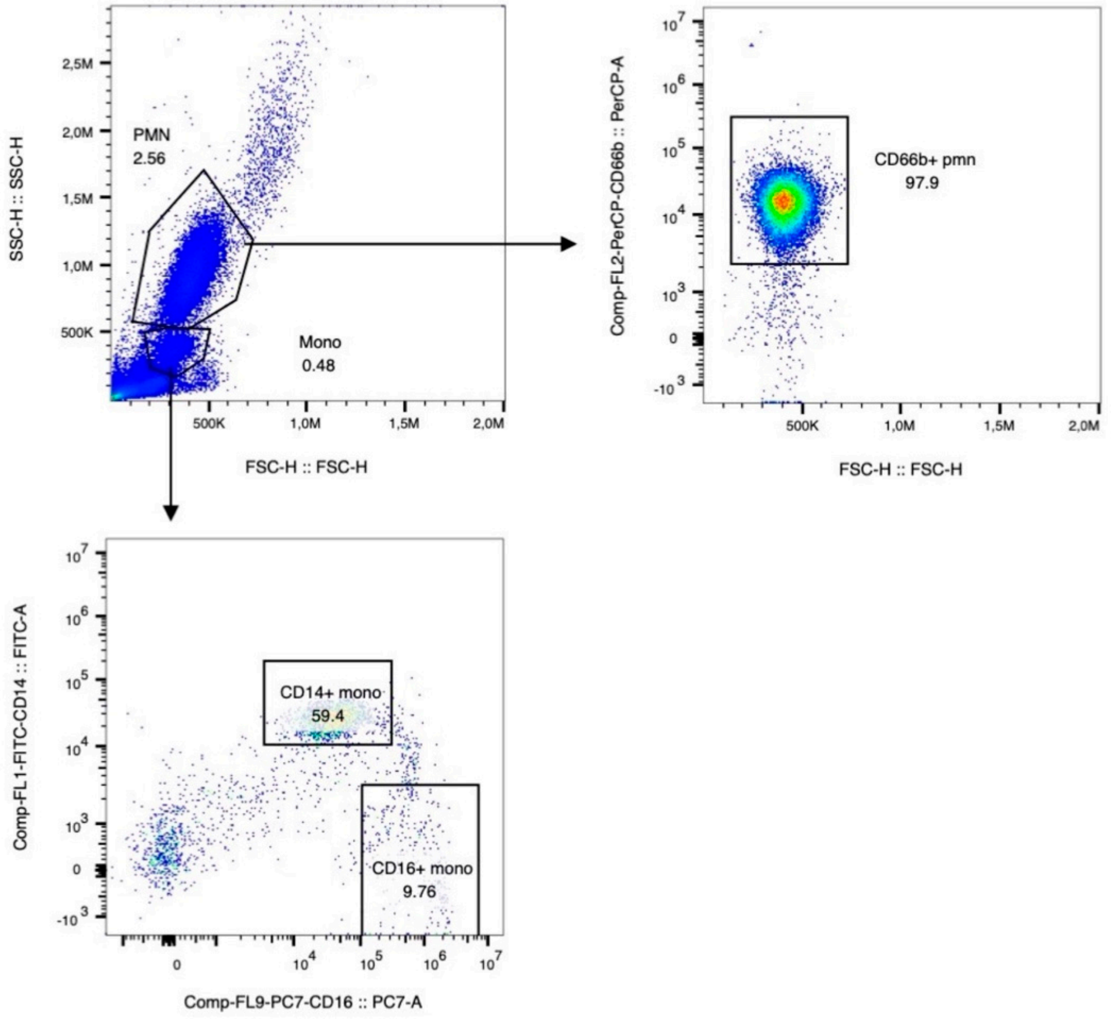

Figure S4. Gating of PMNs and $CD14^+$ monocytes in whole blood. PMNs were defined as FSC^{high}, SSC^{high}, $CD66b^+$ cells and monocytes were defined either as FSC^{medium}, SSC^{medium}, $CD14^+$ cells or FSC^{medium}, SSC^{medium}, $CD16^+$ cells

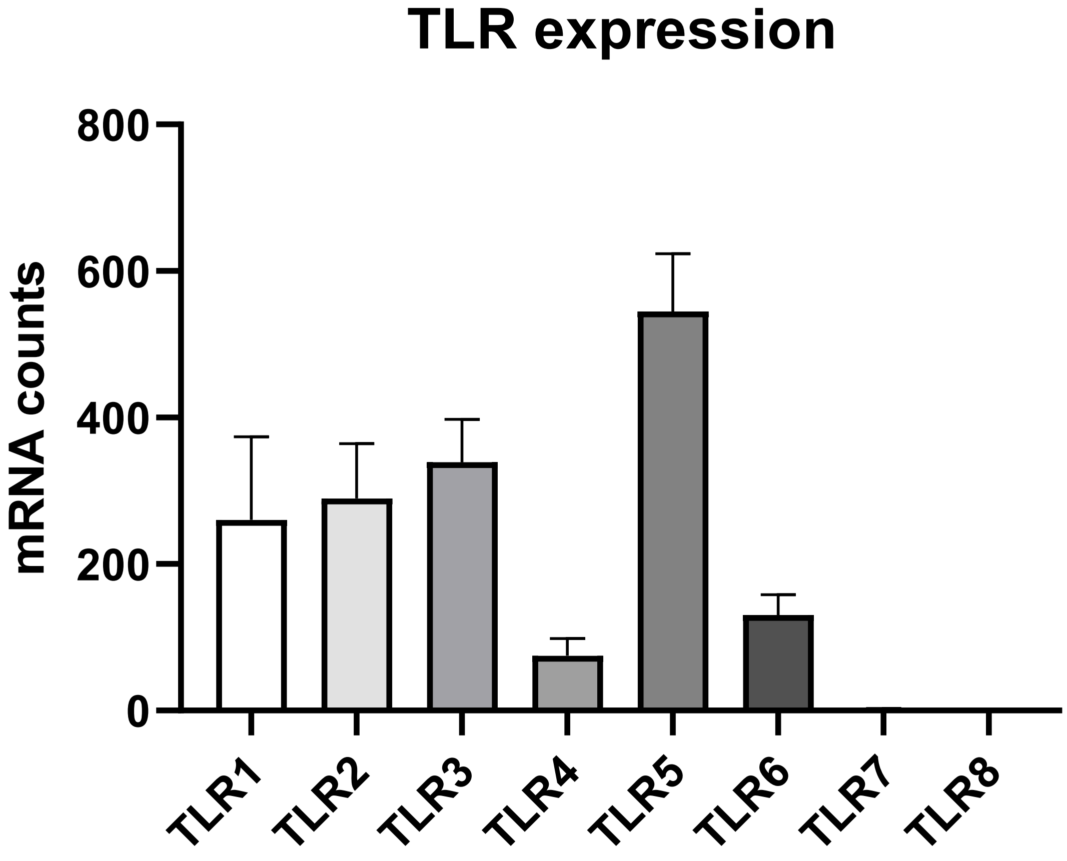

Figure S5. Expression of different Toll-like-receptors (TLRs) in HBE cells. Total mRNA counts of TLRs as determined by RNA sequencing analysis described in reference 12 (accession number for the RNA sequencing data is GEO: GSE164704). Data represent duplicates of unstimulated HBE cells from two different donors.
